# Distributed ultrafast fibre laser

**DOI:** 10.1038/srep09101

**Published:** 2015-03-13

**Authors:** Xueming Liu, Yudong Cui, Dongdong Han, Xiankun Yao, Zhipei Sun

**Affiliations:** 1 State Key Laboratory of Transient Optics and Photonics, Xi'an Institute of Optics and Precision Mechanics, Chinese Academy of Sciences, Xi'an 710119, China; 2 Department of Micro- and Nanosciences, Aalto University, PO Box 13500, FI-00076 Aalto, Finland

## Abstract

A traditional ultrafast fibre laser has a constant cavity length that is independent of the pulse wavelength. The investigation of distributed ultrafast (DUF) lasers is conceptually and technically challenging and of great interest because the laser cavity length and fundamental cavity frequency are changeable based on the wavelength. Here, we propose and demonstrate a DUF fibre laser based on a linearly chirped fibre Bragg grating, where the total cavity length is linearly changeable as a function of the pulse wavelength. The spectral sidebands in DUF lasers are enhanced greatly, including the continuous-wave (CW) and pulse components. We observe that all sidebands of the pulse experience the same round-trip time although they have different round-trip distances and refractive indices. The pulse-shaping of the DUF laser is dominated by the dissipative processes in addition to the phase modulations, which makes our ultrafast laser simple and stable. This laser provides a simple, stable, low-cost, ultrafast-pulsed source with controllable and changeable cavity frequency.

Ultrafast fibre lasers, which play an important role in modern research and industrial applications, have attracted considerable attention because of their compactness, reliability, low cost, and easy turnkey operation^1^,^2^,^3^,^4^,^5^. Passive mode-locking is an efficient way of generating picosecond and femtosecond pulses^6^,^7^. Passively mode-locked (PML) fibre lasers have evolved from fundamental science to commercial instruments, with widespread applications in optical communications, medicine, and materials processing^8^,^9^,^10^,^11^,^12^. The saturable absorber is a key element for the PML fibre lasers. Currently, various saturable absorbers have been proposed, such as the nonlinear polarisation rotation^13^,^14^, nonlinear optical loop mirror^15^,^16^, semiconductor saturable absorber mirror^17^,^18^, graphene^19^,^20^, and single-walled carbon nanotube (SWNT)^21^,^22^. Among them, SWNTs are particularly interesting for ultrafast lasers because they have high environmental stability and are independent of the polarisation of pulses evolving in the laser cavity^21^,^22^,^23^,^24^,^25^.

The most common type of laser cavity is the Fabry–Perot cavity, which is made by placing the gain medium between two high-reflecting mirrors^6^,^26^. The general solution is to deposit dielectric mirrors directly onto the polished ends of a fibre. Another solution is to use fibre Bragg gratings (FBGs) for the mirrors^27^; this has been widely employed in past decades because of its simple design. Recently, linearly chirped fibre Bragg gratings (LCFBGs) have been observed to provide an excessive amount of negative dispersion inside the laser cavity; e.g., a 10-cm-long grating can compensate the dispersion acquired over fibre lengths of 50 km^6^. When the PML fibre laser operates at the fundamental cavity frequency, it delivers a pulse train whose individual pulses are spaced by the round-trip time inside the laser cavity. When the mirror of the laser cavity is distributed (e.g., LCFBG) rather than concentrated (e.g., dielectric mirror), the round-trip distance for different frequencies of pulse is different. Although the distributed lasers can deliver the continuous-wave (CW) lasing^28^,^29^,^30^,^31^, they challenge the mode-locking operation because the frequency spacing among the modes should be constant rather than varied^32^,^33^.

The PML fibre laser with chirped FBG design was first reported in 1995^34^,^35^. This type of linear-cavity laser is widely utilised in modern research and industrial applications because of the all-fibre structure, easy fabrication, and reliability. In general, the LCFBG is employed as a wavelength selection or dispersion management component^34^,^35^,^36^,^37^,^38^,^39^,^40^ so that such lasers typically emit pulses at an individual wavelength with a fixed repetition rate. It is worth noting that the characteristics of the distributed reflection were ignored in previous reports. Thus far, no distributed ultrafast (DUF) phenomenon in fibre lasers has been reported. The pulse-shaping mechanism for DUF fibre lasers is absent because the nonlinear effects in this type of laser are difficult to balance the very large anomalous dispersion induced by the LCFBG.

In this paper, a DUF fibre laser using an LCFBG is proposed and demonstrated experimentally. It differs from the conventionally concentrated ultrafast fibre lasers because the total cavity length of the DUF laser is linearly changeable as a function of the pulse wavelength. Broadband wavelength tuning (from ~1556 to ~1564 nm) is reported, corresponding to ~2 kHz of the tunable range of the fundamental cavity frequency. The spectral sidebands, which are composed of CW and pulse components and are distinct from the Kelly sidebands in the conventional soliton fibre lasers^41^,^43^, are greatly enhanced. We observe that the pulse-shaping of the DUF laser is dominated by the dissipative processes in addition to the phase modulations, which is completely different from the common net-anomalous-dispersion or net-normal-dispersion lasers. Our unique experimental observations are also confirmed by our numerical simulations.

## Results

### Laser set-up and operation

**Figure 1:**
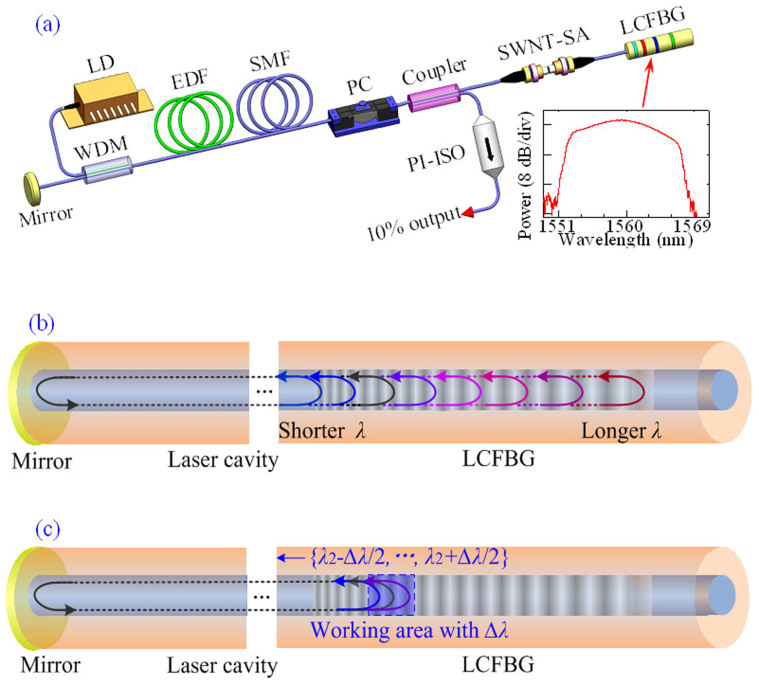
Set-up of DUF fibre laser. (a) Cavity setup of the PML fibre laser incorporating a linearly chirped fibre Bragg grating (LCFBG). Inset: Reflection spectra of LCFBG. The LCFBG is spliced in a linear cavity containing a single-walled carbon nanotube (SWNT) saturable absorber (SA) to mode-lock ultrafast laser, a polarisation controller (PC) to act on the pulse polarisation and adjust the central wavelength, a gain fibre (EDF), a wavelength-division multiplexer (WDM) to couple the pump source (LD), and a high-reflecting dielectric mirror. A polarisation independent isolator (PI-ISO) forces the unidirectional output of the laser. The total length of linear cavity is ~17.7 m with ~7-m-long EDF and ~15-mm-long LCFBG. (b) Schematic diagram of the LCFBG-based fibre laser. The LCFBG reflects the different wavelengths with respect to its position. Round-trip distance for a pulse with a shorter wavelength is less than that for a pulse with a longer wavelength. (c) The operation of ultrafast fibre laser with the central wavelength of *λ*_2_ and the spectral bandwidth of Δ*λ*. The blue area of the LCFBG reflects the spectra from *λ*_2_ − Δ*λ*/2 to *λ*_2_ + Δ*λ*/2. The different spectral components of the pulses propagate through the different distances in a round trip.

The key component of a DUF laser, shown in Figs. 1(a)–1(c), is an LCFBG that introduces a distributed operation for ultrafast pulse generation in this laser. Note that it also provides a large amount of dispersion, up to −5.2 ps^2^/cm (over 10^4^ times larger than the standard fibre), by the concept of the photonic band gap. The experimental set-up is shown in Fig. 1(a) (see Methods for details). The LCFBG is spliced in a standard linear laser cavity. A polarisation controller is employed to control the central wavelength of laser operation by means of the polarisation-dependent loss.

The operational principle of the laser is illustrated in Fig. 1(b). The different parts of the LCFBG reflect the different wavelengths *λ*. The left and right mirrors operate to concentrate and distribute, respectively. The proposed laser cavity is clearly very different from the conventional Fabry–Perot cavity that can provide laser operation with well-defined, equally spaced longitudinal modes. The total length of the laser cavity here is changeable rather than constant when the laser operates on the different wavelengths. As a result, the round-trip distance for different frequencies of a pulse is different. Figure 1(c) illustrates the operation of the ultrafast fibre laser with the spectral range from *λ*_2_ − Δ*λ*/2 to *λ*_2_ + Δ*λ*/2.

### Distributed-operation cavity effect

**Figure 2:**
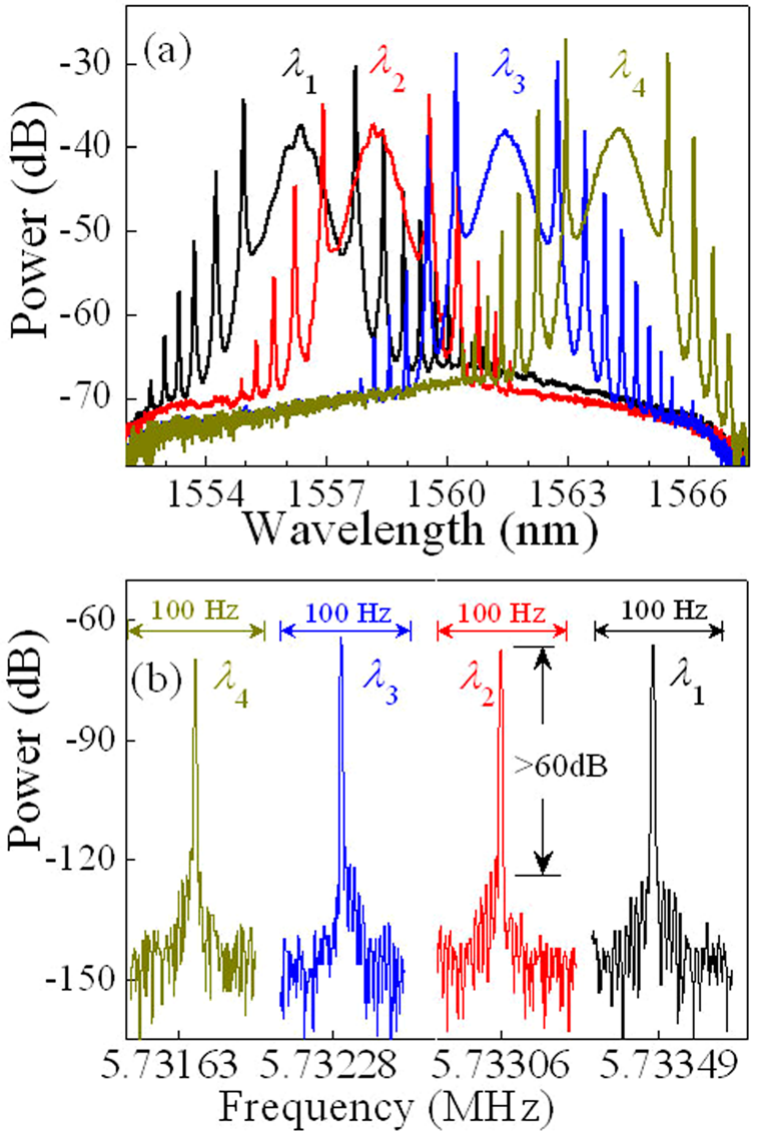
Experimental optical spectra and fundamental radio frequency (RF) spectra of the laser operating on four typical wavelengths. (a,b) The four typical laser outputs at the pump power *P* ≈ 13 mW, achieved through the appropriate adjustment of the polarisation controller, for *λ*_1_ (black), *λ*_2_ (red), *λ*_3_ (blue), and *λ*_4_ (dark yellow). The in-line polarisation controller with low insertion loss can adjust the pulse wavelength by means of the polarisation dependent loss. (a) Optical spectra of the laser at four different wavelengths *λ*_1–4_. The central wavelengths of *λ*_1–4_ are 1556.36, 1558.25, 1561.45, and 1564.25 nm, respectively. (b) Fundamental RF spectra with the resolution of 1 Hz and the span of 100 Hz for the corresponding *λ*_1–4_. The fundamental repetition rates of *λ*_1–4_ are 5.733487, 5.733062, 5.732286, and 5.731641 MHz, respectively.

Self-starting mode-locking operation starts at the pump power of *P* ≈ 10 mW. By appropriately adjusting the settings of the polarisation controller, the proposed laser delivers the pulses with the different central wavelengths and repetition rates. The typical output spectra at *P* ≈ 13 mW are shown in Fig. 2(a), with the central wavelengths *λ*_1–4_ of 1556.36, 1558.25, 1561.45, and 1564.25 nm. The corresponding fundamental cavity frequencies are 5.733487, 5.733062, 5.732286, and 5.731641 MHz, respectively, as shown in Fig. 2(b). The cavity frequency is changed at different wavelengths because the total length of the cavity is automatically adjusted based on the operational wavelength by the LCFBG. This confirms our concept of distributed-operation design. The schematic diagram is demonstrated in Figs. 1(b) and 1(c). If the total length of cavity for *λ*_1_ is *L*, it is approximately *L* + 2.6, *L* + 7.1, and *L* + 11 mm for *λ*_2–4_, respectively. The radio frequency (RF) spectra in Fig. 2(b) give a signal-to-noise ratio of >60 dB (>10^6^ contrast), showing low-amplitude fluctuations and good mode-locking stability^44^.

**Figure 3:**
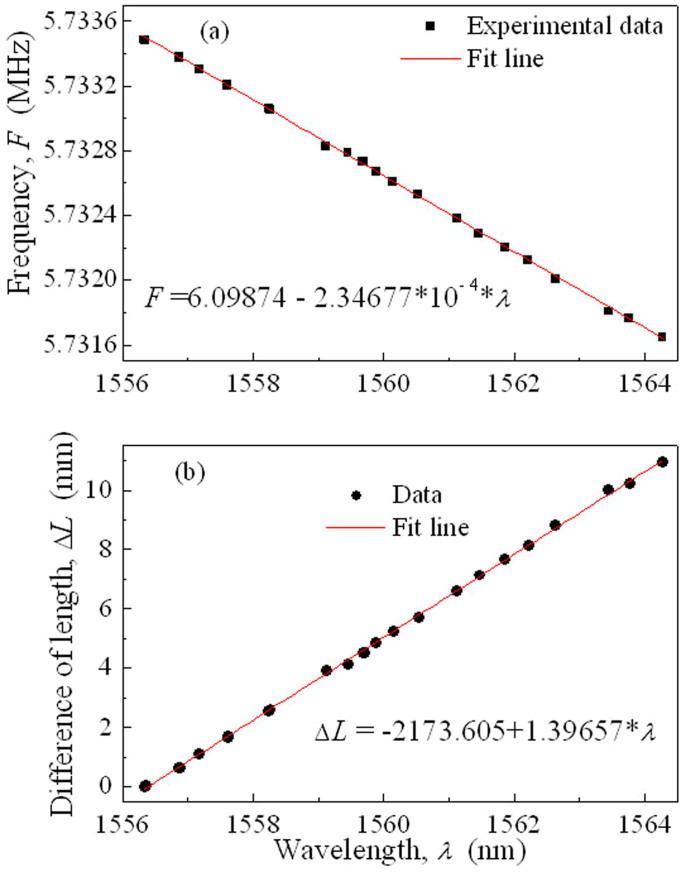
Fundamental cavity frequency and relative cavity length of laser operating on different wavelengths. (a) Fundamental cavity frequency *F* and (b) relative difference of cavity length, Δ*L*, with respect to the central wavelength *λ*. The square symbols are the experimental data, showing the relationship of *F* versus *λ* of pulses. The circle symbols are calculated from the experimental data. In the calculation, Δ*L* is the difference of the total cavity length of any wavelength to a reference wavelength (1556.36 nm). Δ*L* is as large as ~11 mm when *λ* is from ~1556.36 to ~1564.25 nm. The solid lines are fit from the experimental data (square and circle symbols). The fit lines in (a, b) are expressed by *F* = 6.09874 − 2.34677 × 10^−4^·*λ* and Δ*L* = −2173.605 + 1.39657·*λ*, respectively.

Figures 3(a) and 3(b) show the relationships of the fundamental cavity frequency *F* and the relative difference of cavity length, Δ*L*, with respect to the central wavelength *λ*, respectively. The square symbols denote the experimental data and the circle symbols are calculated from the experimental data. The difference of *F*, Δ*F*, is composed of two parts, i.e., Δ*F* = Δ*F*_Cavity_ + Δ*F*_Fiber_. Δ*F*_Cavity_ is from the relative difference of cavity length, Δ*L*, due to the distributed operation. Δ*F*_Fiber_ originates from the group velocity dispersion of intra-cavity (i.e., EDF and SMF in the laser cavity). Here, the dispersions of EDF and SMF in the cavity can cause a frequency difference of ~8 Hz for 1 nm of wavelength difference^45^. The calculation of Δ*L* is given by Δ*L* = *c*Δ*F*_Cavity_/(*nF*^2^), where *n* and *c* are the refractive index of the fibre and the speed of light waves in a vacuum, respectively.

We can observe from Fig. 3(a) that the fundamental cavity frequency *F* decreases approximately linearly with the central wavelength *λ*. The solid lines in Fig. 3 are the fit lines with the expressions of *F* = 6.09874 − 2.34677 × 10^−4^·*λ* and Δ*L* = −2173.605 + 1.39657·*λ*. It is seen from Fig. 3(b) that the difference of the total cavity length, Δ*L*, approximately linearly increases along with *λ*, as interpreted from Fig. 1(b). Because of the linear chirp of the LCFBG, the grating period Λ linearly increases along with the LCFBG (Fig. 1(b)). Then, the reflected wavelength of the grating also increases linearly along with the LCFBG because it is given by 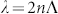. From Fig. 3(b), one can see that Δ*L* is ~ 11 mm when *λ* increases from ~1556.36 to ~1564.25 nm. The corresponding optical and RF spectra for *λ* = 1556.36 and 1564.25 nm are shown in Fig. 2.

### Laser characteristics and theoretical confirmation

**Figure 4:**
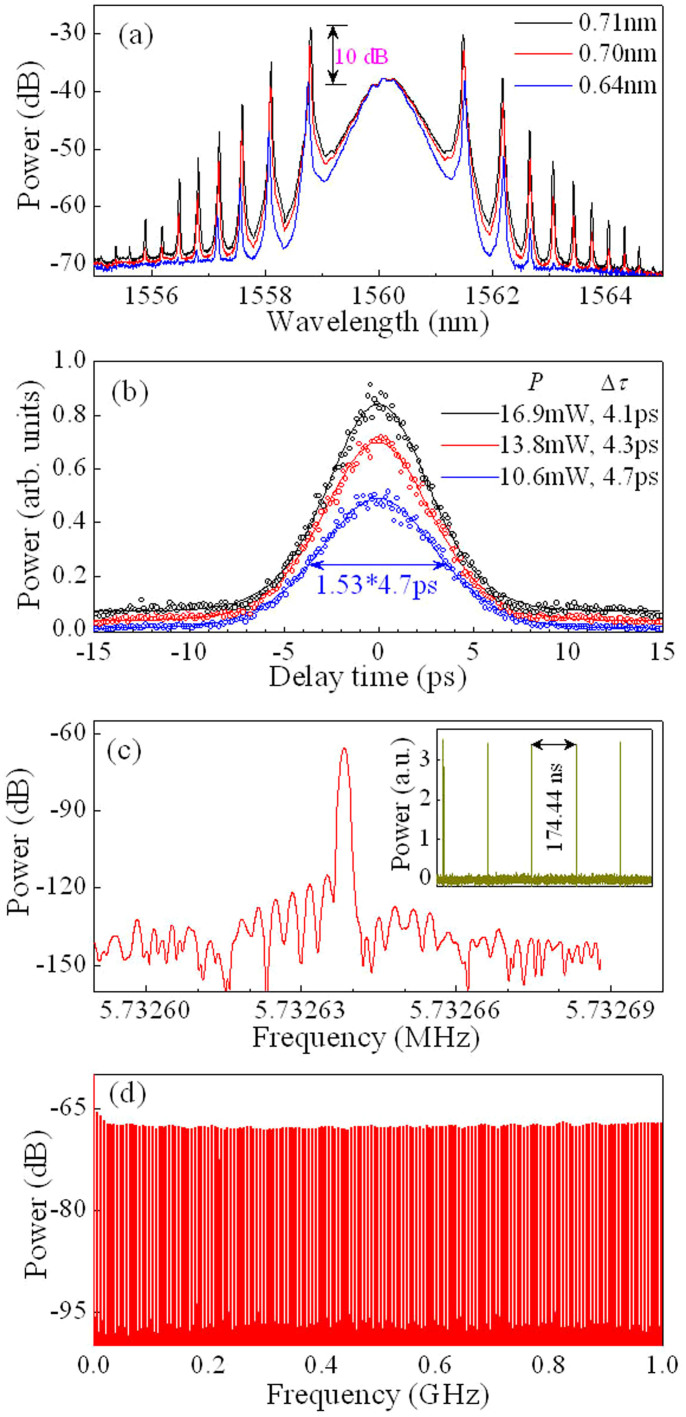
Typical laser characteristics. (a–d) The optical spectra, autocorrelation traces, RF spectra, and oscilloscope traces, respectively, of lasers at the central wavelength *λ* ≈ 1560 nm. (a) Optical spectra of the experimental observations at the pump power *P* = 10.6, 13.8, and 16.9 mW (from bottom to top), respectively. (b) Autocorrelation traces of the experimental data (circle symbols) and sech^2^–shaped fit (solid curves). The FWHM spectral width and the pulse durations (Δ*τ*) are approximately 0.64 nm and 4.7 ps, 0.70 nm and 4.3 ps, and 0.71 nm and 4.1 ps at *P* ≈ 10.6, 13.8, and 16.9 mW, respectively. (c) Fundamental RF spectrum with the resolution of 1 Hz and the span of 100 Hz. Inset: oscilloscope traces with the separation of 174.44 ns, corresponding to 5.732638 MHz of the fundamental harmonic frequency that is independent of the pump power. (d) Wideband RF spectrum up to 1 GHz. No spectrum modulation is observed over 1 GHz in (d), indicating no Q-switching instabilities.

Figures 4(a)–4(d) show the optical spectra, autocorrelation traces, RF spectra, and oscilloscope traces respectively of lasers at *λ* ≈ 1560 nm. The typical output spectra at the pump powers of *P* ≈ 10.6, 13.8, and 16.9 mW are shown in Fig. 4(a). The corresponding autocorrelation traces of the experimental data (circle symbols) and the sech^2^–shaped fit curve are shown in Fig. 4(b). The full width at half maximum (FWHM) spectral width and the pulse durations (Δ*τ*) are approximately 0.64 nm and 4.7 ps, 0.70 nm and 4.3 ps, and 0.71 nm and 4.1 ps at *P* ≈ 10.6, 13.8, and 16.9 mW, respectively. Then, the corresponding time-bandwidth products are approximately 0.37, 0.37, and 0.36, respectively, which are slightly larger than the value of 0.315 for the transform-limited sech^2^-shaped pulses. Figures 4(c) and 4(d) are the fundamental RF spectra with 1 Hz resolution and 100 Hz span and the wideband RF spectra up to 1 GHz, respectively. Figure 4(c) demonstrates that the repetition rate of the fundamental harmonic frequency is 5.732638 MHz, corresponding to 174.44 ns round-trip time (Fig. 4(c) inset). No spectrum modulation is observed over 1 GHz (Fig. 4(d)), indicating no Q-switching instabilities.

The experimental observations show that with the increase of the pump power *P*, the optical spectrum is hardly improved for the central wavelength whereas it is evidently enhanced for the sidebands. An example is shown in Fig. 4(a). At the same time, the pulse energy increases along with *P*, as shown in Fig. 4(b). We can see from Fig. 4(a) that when *P* increases from 10.6 to 16.9 mW, the sideband is improved by ~10 dB (i.e., 10 times) although the spectral power at the central wavelength (i.e., ~1560 nm) is hardly changed. The experimental results show that the maximum of the output average power of pulses is approximately 0.6 mW at *P* ≈ 22 mW for the single pulse operation of the laser, corresponding to the pulse energy of ~1 nJ in the intracavity. When the pump power *P* is beyond 22 mW, the laser operates on the dual-pulse regime.

**Figure 5:**
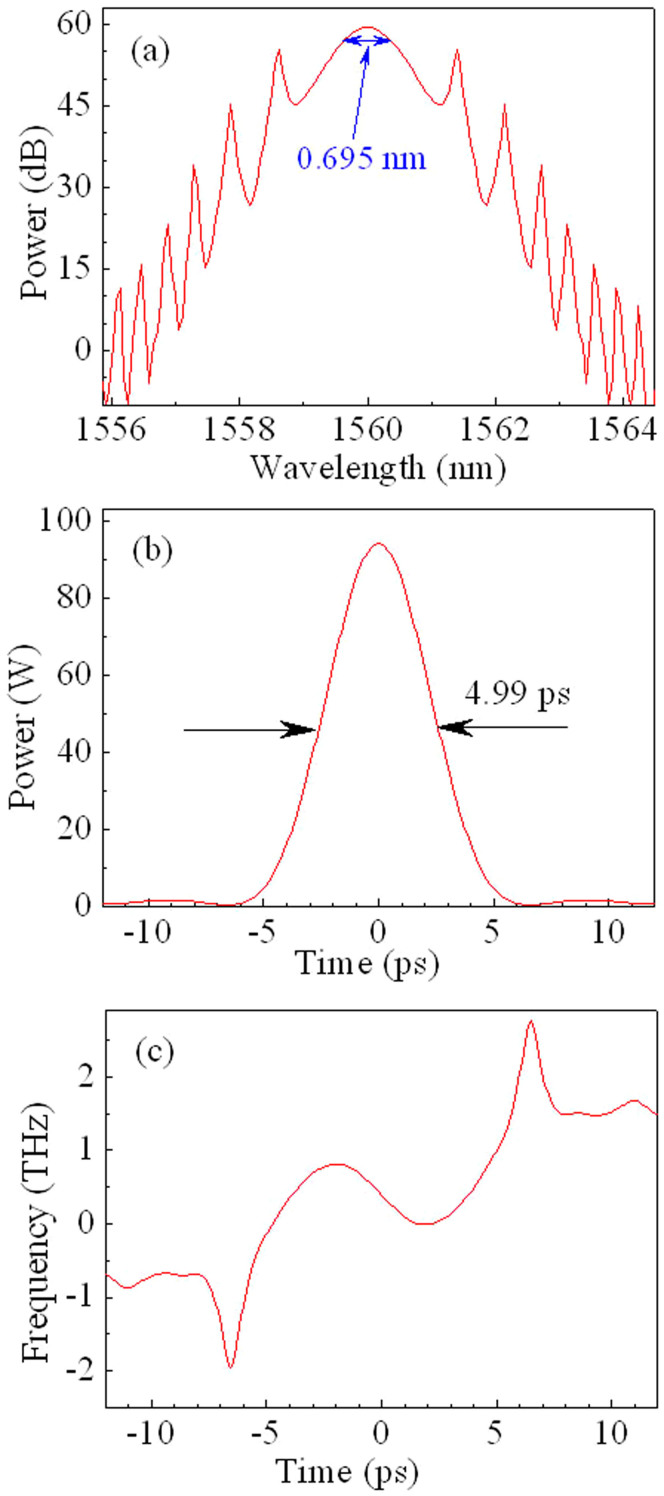
Numerical simulations. To match the experimental results, the pump strength *E_s_* is assumed to be 135 pJ in the calculation. The pulse has 0.695 nm of the FWHM spectral width, 4.99 ps of the pulse duration, and 0.5 nJ of pulse energy. The numerical result is in good agreement with the experimental observation in Fig. 4 (at the case of the pump power *P* = 10.6 mW). (a) Optical spectrum, (b) pulse profile, and (c) instantaneous frequency of the pulses.

To confirm the experimental observations, the typical results of numerical simulations of laser in the mode-locking regime are demonstrated in Fig. 5. In the modelling, in addition to the phase modulation, the dissipative processes (i.e., gain and loss processes^46^) play a crucial role in driving the system to the steady-state solution. Note that the spectral filtering effect is ignored in the simulations. Parameters are chosen to match the experimental values (see Methods). It is seen from Fig. 5 that the spectral width and pulse duration are 0.695 nm and 4.99 ps, respectively. So the time-bandwidth product is approximately 0.43, showing that it is sech^2^-shaped pulses rather than Gaussian-shaped pulses. The pulse energy is approximately 0.5 nJ, which can be enhanced by increasing the pump strength *E_s_*. The numerical results (Figs. 5(a) and 5(b)) are in good agreement with the experimental observations, as shown in Fig. 4 (at the case of the pump power *P* = 10.6 mW). Figure 5(c) shows that the instantaneous frequency is low and nonlinear across the pulse. From the theoretical point of view, then, the pulses are hardly compressed and dechirped.

We can observe from Fig. 3(b) that the length of the working area of the LCFBG, δ*L*, is ~ 1.39 mm for 1 nm of wavelength difference. Then, the delay related to the LCFBG is ~ 4.7 ps when the FWHM spectral width of the pulse is 0.7 nm. This delay is approximately consistent with the pulse duration, as shown in the experimental and theoretical results (Figs. 4 and 5). Therefore, the spectral and temporal widths of pulses in the DUF lasers are dependent on the relative differences of cavity length.

### Strong enhancement of spectral sidebands

Usually, no clear evidence of Kelly sidebands has emerged in stretched-pulse lasers^47^, self-similar lasers^48^, dissipative-soliton lasers^49^, and graded-index multimode fibre lasers^50^. By contrast, at the phase-matched frequencies of soliton lasers, the dispersive radiation builds up and causes Kelly sidebands on the spectrum. However, the Kelly sideband creation is a key limitation on the soliton energy of lasers^51^. Then, the pulse energy of a conventional soliton is typically less than 0.1 nJ in the standard fibre^52^,^53^. The theoretical predictions and experimental observations show that the pulse energy in this report can be up to 1 nJ.

**Figure 6:**
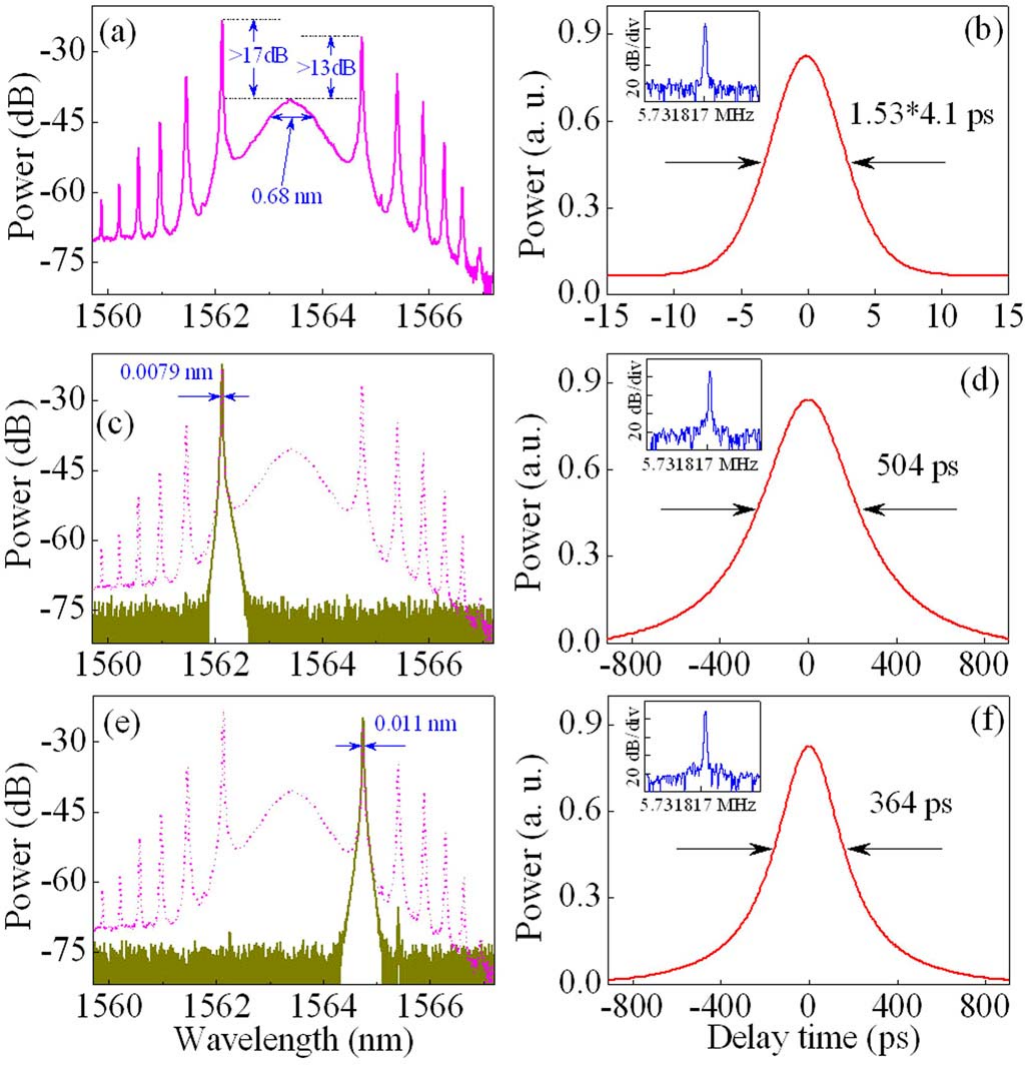
Strongly enhanced sidebands. The laser output at the pump power *P* ≈ 17 mW with the appropriate setting of polarisation controller. (a) Optical spectrum and (b) the corresponding autocorrelation trace of laser. The central wavelength of the pulse is ~1563.4 nm. The two strongest sidebands at the wavelengths of ~1562.1 and ~1564.7 nm are over 17 and 13 dB larger than the central wavelength, respectively. The laser pulse has 0.68 nm of the FWHM spectral width, 4.1 ps of the pulse duration, and 5.731817 MHz of fundamental cavity frequency (Fig. 6(b) inset). (c, e) The two strongest sidebands are separated from the pulse spectrum by a programmable optical filter with the bandwidth of 0.3 nm. Optical spectrum (c) and autocorrelation trace (d) of the strongest sideband at ~1562.1 nm. Optical spectrum (e) and autocorrelation trace (f) of the second strongest sideband at ~1564.7 nm. Inset: Fundamental RF spectrum with the resolution of 1 Hz and the span of 100 Hz for the pulse (Fig. 6(b)), the strongest sideband (Fig. 6(d)), and the second strongest sideband (Fig. 6(f)). The two strongest sidebands have different wavelengths with the difference of 2.6 nm, but they have the same round-trip time of 174.46475 ns (i.e., reciprocal of 5.731817 MHz of fundamental harmonic frequency).

The strongest peak of sidebands and the spectral power of the central wavelength are of the same order of magnitude for conventional soliton lasers^41^,^42^,^43^,^51^. However, the experimental results here demonstrate that the power of the first-order sidebands is much stronger than that of the central wavelength, as shown in Figs. 2(a), 4(a), and 6(a). We can observe from Fig. 6(a) that the two strongest sidebands are over 17 dB (i.e., ~50 times) and 13 dB (i.e., ~20 times) larger than the spectral power of the central wavelength (~1563.4 nm), respectively. The experimental observations show that, with the increase of pump power, the pulse energy and sidebands are enhanced but the power at the central wavelength is almost unchanged. By comparing the experimental observations (i.e., Fig. 4(a)) to the theoretical results (i.e., Fig. 5(a)), we can see that, for lower pump strength, the experimental results are in good agreement with the theoretical predictions. For higher pump strength, but, the power of the first-order sideband is much larger than that of the central wavelength, e.g., the former is 50 times larger than the latter (Fig. 6(a)). The modulation instability plays the key role for the higher pump strength.

The strongest two sidebands in Fig. 6(a) are separated from the pulse spectrum by a programmable optical filter. The solid curves in Figs. 6(c) and 6(e) are the separated sidebands with the spectral widths of 0.0079 and 0.011 nm, respectively. Figures 6(d) and 6(f) illustrate the autocorrelation traces of spectral sidebands at 1562.1 and 1564.7 nm, indicating that the FWHM widths are ~504 and ~364 ps, respectively. They are much larger than the pulse duration of the laser (Fig. 6(b)).

## Discussion

Fibre dispersion of the laser cavity plays a critical role in the evolution of pulses because different spectral components associated with the pulse travel at different speeds. Usually, the net-anomalous-dispersion fibre lasers support solitons through a balance between the dispersive and nonlinear effects^54^. In the large normal dispersion lasers that have no intra-cavity dispersion control, the spectral filtering produces strong self-amplitude modulation that can dominate the pulse-shaping^13^,^53^, which is qualitatively distinct from the soliton-like processes. The above-mentioned lasers have a constant cavity length so that each spectral component of the pulse propagates the same distance. By contrast, the fibre lasers with the distributed mirrors have different distances for different spectral components of the pulse. Both experimental observations and theoretical results show that the gain of the laser plays a critical role in the steady-state pulses. Therefore, the pulse-shaping in the DUF fibre lasers dominates from the gain and loss processes (i.e., dissipative processes) in addition to the phase modulations, which is different from the large normal dispersion lasers where the spectral filtering effect plays a key role. In addition, the spectral width of the DUF laser is less than 1 nm, which is much narrower than that of large normal dispersion lasers^13^,^53^.

Figure 6 shows that although the two separated sidebands have different wavelengths with a difference of 2.6 nm, their fundamental harmonic frequencies are the same as the fundamental cavity frequency (i.e., 5.731817 MHz). In fact, the experimental observations show that all sidebands in Fig. 6(a) have the same fundamental harmonic frequency, i.e., the same round-trip time. Note that different sidebands have different round-trip distances that are determined by the LCFBG. By contrast, although the right and left third-order sidebands of *λ*_1_ and *λ*_3_ in Fig. 2(a) have approximately the same wavelength (i.e., ~1559 nm), they have different fundamental cavity frequencies, i.e., 5.733487 and 5.732286 MHz, respectively.

**Figure 7:**
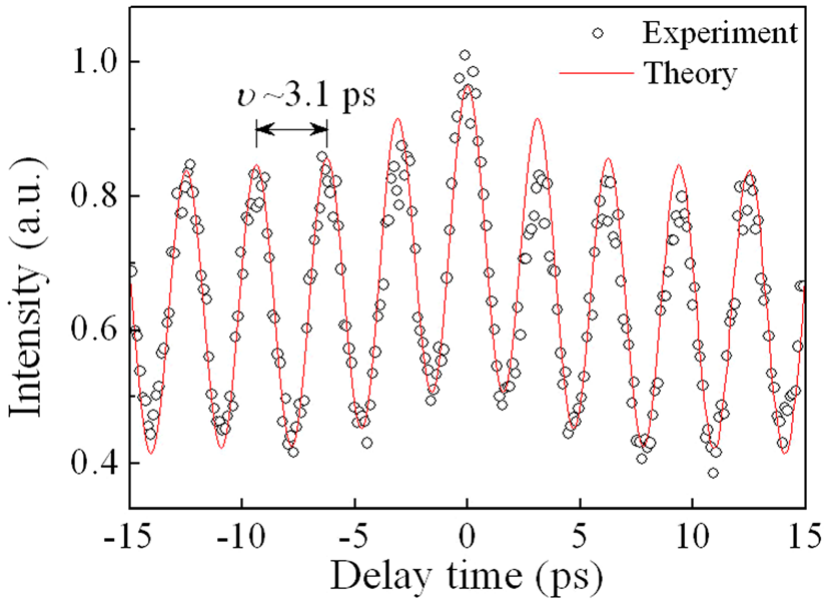
Autocorrelation traces of interaction of the first-order sidebands. The red solid curve and circular symbols are the theoretical and experimental results, respectively. The period of curve, *ν*, is approximately 3.1 ps. *ν* is equal to the reciprocal of the wavelength difference (i.e., 2.6 nm) of the first-order sidebands. Note that 2.6 nm of the wavelength difference corresponds to 320 GHz of the frequency difference.

To check the coherence of sidebands, we filter the first-order sidebands from the optical spectrum in Fig. 6(a). In experiments, the first-order sidebands (i.e., the strongest two sidebands in Fig. 6(a)) are almost unchanged but the other part of the spectrum is attenuated by >20 dB. Their autocorrelation traces are shown in Fig. 7. The solid curve and symbols are the theoretical and experimental results, respectively. It is seen from Fig. 7 that the period of the curve, *ν*, is approximately 3.1 ps, which is equal to the reciprocal of the difference of the first-order sidebands (i.e., 320 GHz of the frequency difference corresponds to ~2.6 nm of the wavelength difference).

The spectral sidebands result from the resonant enhancement of certain frequencies of dispersive waves that are the low-level and broadband background^6^. Here, the experimental observations reveal that the spectral sidebands in DUF lasers have the pulse behaviours, as shown in Figs. 6 and 7. To better understand the spectral sidebands, they are measured by an oscilloscope (see Supplementary Material). The experimental results show that the spectral sidebands contain the CW and pulse components. The strongest spectral sideband includes approximately 51% of the pulse component (see Supplementary Material).

In conclusion, we have proposed a DUF laser from the conceptual point of view. The experimental results show that the total cavity length of the DUF fibre laser is linearly changeable as a function of the pulse wavelength, different from the conventional concentrated ultrafast fibre lasers. The spectral sidebands, which include CW and pulse components, in DUF fibre lasers are enhanced greatly. All of the sidebands have the same round-trip time although they have different round-trip distances and refractive indices. The bandwidth of the first-order sidebands is as narrow as ~0.008 nm. The pulse-shaping of the DUF fibre laser dominates from the dissipative processes in addition to the phase modulations, which is different from the common net-anomalous-dispersion or net-normal-dispersion lasers. The theoretical simulations are in good agreement with the experimental observations.

## Methods

### Set-up and experiments

The experimental setup for the laser cavity is shown in Fig. 1(a). The conceptual model of the fibre laser is presented in Fig. 1(b). The laser system consists of a high-reflection dielectric mirror, a wavelength-division multiplexer (WDM), a polarisation controller (PC), a fused coupler with 10% output ratio, a single-wall carbon nanotube (SWNT) saturable absorber (SA), an LCFBG, a 7-m-long erbium-doped fibre (EDF) with 6 dB/m absorption at 980 nm, and a segment of standard single-mode fibre (SMF). The EDF provides the gain amplification for the laser system pumped by a 977-nm laser diode (LD). A polarisation independent isolator (PI-ISO) is used to ensure the unidirectional transmission of the laser output. The total length of the linear laser cavity is approximately 17.7 m. The EDF and SMF have dispersion parameters of about −9 and 17 ps/(nm·km) at 1550 nm, respectively. The LCFBG, written on a standard SMF, has a super-Gaussian reflection profile with a bandwidth of ~15 nm (Fig. 1(a) inset). The dispersion parameter of the LCFBG is about −5.2 ps^2^/cm with a length of ~15 mm and a central wavelength of ~1560 nm. The integrated SWNT-based fibre device is realised by sandwiching a ~2 mm^2^ sample between two fibre connectors, as shown in detail in our previous reports^21^.

### Measurement method

An optical spectrum analyser (Yokogawa AQ-6370), an ultra-high resolution optical spectrum analyser (APEX AP2041B), an autocorrelator, a 6-GHz oscilloscope, a radio-frequency (RF) analyser, and a 10-GHz photodetector are used to measure the laser output performances.

### Numerical simulation

To confirm the pulse characteristics, we numerically simulate the pulse formation of the proposed laser. The numerical modelling includes the physics terms such as the group velocity dispersion of fibre, the self-phase modulation, the dispersion of the LCFBG, and the gain of the EDF. Because the spectral width of the pulses is much narrower than that of the gain and the LCFBG, the spectral filtering effect is ignored in the modelling. Therefore, we use the nonlinear Schrödinger equation to describe the pulse propagation in the laser oscillator^54^, i.e.,
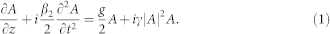
Here *A*, *β*_2_, and *γ* denote the electric field envelope of the pulse, the fibre dispersion, and the cubic refractive nonlinearity of the fibre, respectively. The variables *t* and *z* represent the time and the propagation distance, respectively. *g* describes the gain function of the EDF and is expressed by *g* = *g*_0_·exp(−*E_p_*/*E_s_*)^55^,^56^, where *g*_0_, *E_p_*, and *E_s_*are the small-signal gain coefficient related to the doping concentration, the pulse energy, and gain saturation energy that relies on pump power, respectively. The theoretical modelling here is different from that in the previous reports^21^,^22^, i.e., the spectral filtering term is excluded in Eq. (1), whereas it is included in the latter.

Based on a two-level saturable absorber model^21^,^57^, the intensity-dependent absorption coefficient is given by *α*(*I*) = *α*_ns_ + *α*_0_/(1 + *I*/*I*_sat_), where *α*_0_, *α*_ns_ and *I*_sat_ are the linear limit of saturable absorption, nonsaturable absorption, and saturation intensity, respectively.

To numerically simulate the properties and behaviour of the laser, the simulation is started from an arbitrary signal and converges to a stable solution after approximately 100 round trips. In the simulation, we use the following parameters to match the experimental conditions: *g*_0_ = 6 dB/m, *E_s_* = 135 pJ, *β*_2_ = 11 ps^2^/km and *γ* *= * 1.8 W^−1^km^−1^ for EDF, and *β*_2_ = −22 ps^2^/km and *γ* *= * 1 W^−1^km^−1^ for SMF. The parameters of SWNT-SA are set with the values measured^21^, i.e., *α*_0_ = 12.05%, *α*_ns_ = 87.87%, and *I*_sat_ = 9.67 MW/cm^2^.

## Author Contributions

X.L. proposed the laser system, completed the numerical simulation, and wrote the main manuscript text. Y.C. performed the main experimental results. D.H. performed part simulation. X.Y. prepared part figures. Z.S. contributed to the scientific discussion. All authors discussed the results and substantially contributed to the manuscript.

## Supplementary Material

Supplementary Information Distributed ultrafast fibre laser
