# Supplementary material for: Distributed ultrafast fibre laser
Source: Sci Rep. 2015 Mar 13;5:9101. doi: 10.1038/srep09101 (PMC4358043; doi:10.1038/srep09101)
Supplement: Supplementary Information — Distributed ultrafast fibre laser [file srep09101-s1.doc]

**Supplementary material**

**Distributed ultrafast fibre laser**

Xueming Liu^1,*^, Yudong Cui^1^, Dongdong Han^1^, Xiankun Yao^1^, & Zhipei Sun^2^

^1^State Key Laboratory of Transient Optics and Photonics, Xi’an Institute of Optics and Precision Mechanics, Chinese Academy of Sciences, Xi’an 710119, China

^32^ Department of Micro- and Nanosciences, Aalto University, PO Box 13500, FI-00076 Aalto, Finland

*Corresponding author: [liuxueming72@yahoo.com](mailto:liuxueming72@yahoo.com)

**Supplementary note 1: the pulse and CW components in sidebands**

Generally, the sidebands are considered as the dispersive wave shed from solitons. However, the behavior of sidebands in the time domain is not very clear. The photodiode-oscilloscope system is usually utilized to monitor the temporal feature, and the background energy is observed on oscillogram [1]. Moreover, the fast development on photodiode and oscilloscope provides higher resolution and larger dynamic range. The output light included pulses and background energy can be transformed into the electronic signal by a fast photodiode without capacitors blocking the direct current (DC) component. As shown in Supplementary Fig. S1, the electronic pulse corresponds to the optical pulse component and the value of the base line with respect to the zero voltage represents the background energy. The rise time of the photodetector is about 0.3 ns, which is not allowed to measure the intensity of the ultrashort pulse as a function of time, while the pulse energy is measured. On the other hand, the level of the background energy can be measured directly.

**Supplementary Figure S1:** the diagram of pulse and background energy in a pulse train. The pulse above the base line is the pulse component. The value of the base line with respect to the zero voltage represents the background energy.

**Supplementary Figure S2:** (a) The oscilloscope trace of a pulse train. (b) Zoomed-in oscilloscope trace around the (red) box in (a). (c) Zoomed-in oscilloscope trace around the (blue) box in (a). The red cure in (c) is the data without signal.

Similar to Fig. 6(c) in the manuscript, the left first-order sideband is filtered and monitored with an oscilloscope. Under the average mode, an average value of multiple measurements is directly obtained from the oscilloscope, which minimizes the influence of the random noise. Supplementary Fig. S2 shows the averaged oscilloscope trace. By comparing to the data without signal (0.52 mV), the voltage with signal (1 mV) is shifted up. The background energy is about 0.48 mV which has been excluded the noises induced by the oscilloscope and the photodiode, and the peak of the electronic pulse is ~290 mV. The photocurrent ratio *r* of the pulse peak and background is ~600. Supplementary Refs. [1] and [2] provide a method of estimating the energy ratio *θ* of the pulse and total energy from the oscilloscope trace. The photocurrent ratio *r* can be expressed as [2],

$r=\frac{j_{p}}{j_{b}}=\frac{(\kappa\Delta t/T_{d})I_{p}}{\kappa I_{b}}=\frac{\Delta t}{T_{d}}\cdot\frac{m\eta^{2}}{1-\eta}$ (1)

Here *j_p_* and *j_b_* represent photocurrent values of the pulse peak and the background energy, respectively. *I_p_* and *I_b_* are the intensity of the pulse peak and the background energy. *κ* is the quantum efficiency of the photodiode. Δ*t* is the pulse width and *T_d_* is the rise time of the photodetector. *η* is the ratio of the number of locked mode and the number of the all the oscillating mode, which is employed in a partial locking model. *m*=Δ*ν* /ΔΩ is the number of all the oscillating modes, where Δ*ν* is the spectral bandwidth and ΔΩ is the mode separation. From equation (1), the value of *η* can be obtained with *r*. As shown in Supplementary Ref. [2], the peak-to-background contrast ratio *R* of the oscilloscope trace can be calculated from *η*, i.e.,

$R=\frac{3(1+\zeta/m)}{1+2\zeta/m},$ $\zeta=\frac{3(1-\eta^{2})}{\eta}\frac{2m^{2}}{2(m\eta)^{2}+1}.$ (2)

The relationship between the contrast ratio *R* and the energy ratio *θ* is shown in Supplementary Ref. [1], i.e.,

$R=\frac{3+6(1/\theta^{2}-1)(T_{d}/t)\alpha}{1+4(1/\theta^{2}-1)(T_{d}/t)\alpha},$ (3)

where the form factor *α* is 1.51 for Gaussian pulse [1]. So the energy ratio *θ* can be calculated from the photocurrent ratio *r* of the oscilloscope trace. In the experiments, Δ*t*=364 ps, *t*=178 ns, *T_d_* =0.3 ns, Δ*ν* = 1.36 GHz for sideband, and ΔΩ = 5.73 MHz. After some manipulation, the energy ratio *θ* is calculated to be ~ 0.51. Therefore, the left first-order sideband includes about 51% of the pulse component and about 49% of other components (e.g., CW and noise).

**Supplementary Reference**

1. D. von der Linde, “Experimental study of single picosecond light pulses,” IEEE J. Quantum Electron. QE-8, 328 (1972).
2. R. H. Picard and P. Schweitzer, “Theory of intensity-correlation measurements on imperfectly mode-locked lasers,” Phys. Rev. A **1**, 1803-1819 (1970).
3. M. L. Dennis, I. N. Duling III, “Experimental study of sideband generation in femtosecond fiber lasers,” IEEE J. Quantum Electron. **30**, 1469-1477 (1994).
4. J. P. Gordon, “Dispersive perturbations of solitons of the nonlinear Schrodinger equation,” J. Opt. Soc. Am. B **9**, 91-97 (1992).
